# Electrophysiological and Behavioral Responses of Male Fall Webworm Moths (*Hyphantria cunea*) to Herbivory-Induced Mulberry (*Morus alba*) Leaf Volatiles

**DOI:** 10.1371/journal.pone.0049256

**Published:** 2012-11-14

**Authors:** Rui Tang, Jin Ping Zhang, Zhong Ning Zhang

**Affiliations:** 1 State Key Laboratory of Integrated Management of Pest Insects and Rodents, Institute of Zoology, Chinese Academy of Sciences, Beijing, People's Republic of China; 2 Graduate University of the Chinese Academy of Sciences (GUCAS), Beijing, People's Republic of China; Ghent University, Belgium

## Abstract

Volatile organic compounds (VOCs) were collected from damaged and intact mulberry leaves (*Morus alba* L., Moraceae) and from *Hyphantria cunea* larvae by headspace absorption with Super Q columns. We identified their constituents using gas chromatography-mass spectrometry, and evaluated the responses of male *H. cunea* antennae to the compounds using gas chromatography-flame ionization detection coupled with electroantennographic detection. Eleven VOC constituents were found to stimulate antennae of male *H. cunea* moths: β-ocimene, hexanal, cis-3-hexenal, limonene, trans-2-hexenal, cyclohexanone, cis-2-penten-1-ol, 6-methyl-5-hepten-2-one, 4-hydroxy-4-methyl-2-pentanone, trans-3-hexen-1-ol, and 2,4-dimethyl-3-pentanol. Nine of these chemicals were released by intact, mechanically-damaged, and herbivore-damaged leaves, while cis-2-penten-1-ol was released only by intact and mechanically-damaged leaves and β-ocimene was released only by herbivore-damaged leaves. Results from wind tunnel experiments conducted with volatile components indicated that male moths were significantly more attracted to herbivory-induced volatiles than the solvent control. Furthermore, male moths' attraction to a sex pheromone lure was increased by herbivory-induced compounds and β-ocimene, but reduced by cis-2-penten-1-ol. A proof long-range field trapping experiment showed that the efficiency of sex pheromone lures in trapping male moths was increased by β-ocimene and reduced by cis-2-penten-1-ol.

## Introduction

The fall webworm, *Hyphantria cunea* (Drury) (Lepidoptera: Arctiidae), is native to North America but spread into central Europe and eastern Asia in the 1940s [1]. In China, this species was first found in Liaoning Province in 1979 and has since been introduced into other regions, such as Beijing, Tianjin, and Hebei, Shandong, and Shanxi provinces [2]. This polyphagous defoliator has caused huge damage to forests, urban ornamental trees, and agricultural crops in China because of its wide host range [2]. Currently, 175 different plant species within 49 families and 108 genera are known to serve as its hosts [3]. More than 30 years after its initial invasion, *H. cunea* is now widely distributed in eastern and northeastern China. The major hosts of *H. cunea* in China are mulberry, poplar and rock maple, which are all different from those in their native area [4]. Mulberry is especially prone to herbivory by *H. cunea*, because of the plant's high density in China for silkworm breeding and agriculture.

Some of the volatile organic compounds (VOCs) released by herbivore-attacked plants are known to attract arthropod predators and parasitoids in laboratory experiments on agricultural plants [5] and have been shown to function defensively under natural conditions [6]. In some cases, host-related plant volatiles, such as trans-2-hexenal, benzaldehyde, 1-hexenol, cis-3-hexen-1-ol, geraniol, linalool, (+)-limonene, hexenal, and trans-2-hexenol, could increase the sensitivity of insect antennae towards pheromones [7]. Research with *H. cunea* showed that previous herbivory on red alder may improve food quality for fall webworm larvae [8]. On the other hand, eastern tent caterpillar infestations on black cherry negatively affect fall webworm use of the same trees [9]. Although mulberry volatiles have been identified by some researchers [10]–[13], the relationship between mulberry VOCs and *H. cunea* remains unreported. The aims of this study were to identify the bioactive compounds of mulberry leaf volatiles by electrophysiological analysis with *H. cunea* male moths and to identify blends of volatile emissions with potential effects on pheromone trapping.

## Materials and Methods

### Moths

Insects were provided by the Chinese Academy of Forestry, which established a laboratory population of *H. cunea* using wild moths collected in Qinghuangdao, Hebei Province, China. Pupas were kept at 25±1°C under a 16 h light/8 h dark photoperiod in environmental chambers for one week before eclosion. Third-instar larvae were starved for one day before the experiment.

### Headspace collection of VOCs

Chemicals used in all experiments are shown in Table 1. All glassware was carefully cleaned, washed with acetone, and heated at 200–230°C overnight before use. The Super Q GC Packed Column (80/100, Alltech, Deerfield, IL, USA) used to collect volatiles in air entrainment experiments was washed with acetone, tested for purity by gas chromatography (GC), and then conditioned by heating overnight in a stream of nitrogen at 100°C.

**Table 1:** List of chemicals, and their purity and source, used in the experiments.

Chemicals	Purity%	Source*
Pentane	Analytically pure**	a
Diethyl ether	Analytically pure**	b
Acetone	Analytically pure**	b
β-ocimene	95%	c
Limonene	95%	d
trans-2-hexenal	99%	e
trans-3-hexen-1-ol	97%	f
Hexanal	95%	c
cis-2-penten-1-ol	95%	d
cis-3-hexenal	95%	c
4-hydroxy-4-methyl-2-pentanone	99+%	g
2,4-dimethyl-3-pentanol	99%	d
6-methyl-5-hepten-2-one	98%	d
Cyclohexanene	99.80%	e

* a: Shantou Xilong Chemical Factory, Guangdong, PRC; b: Beijing Chemical Factory, Beijing, PRC; c: Sigma-Aldrich, St. Louis, MO, USA; d: Tokyo Chemical Industry Co., Tokyo, Japan; e: J&K Chemical, Shanghai, PRC; f: Alfa Aesar, Ward Hill, MA, USA; g: Acros Organics, New Jersey, USA.

** tested with GC.

Mulberry plants were bought from Jiangsu Province and then grown in laboratory conditions with 25°C and 30% relative humidity for 1 month before experiments in order to make them stable. Fully grown, intact plants at a height of 1.2–1.3 m were chosen. Volatiles were collected by encasing all upper parts of each plant in oven bags (turkey size, Reynolds, Richmond, Virginia, USA) during absorption.

We conducted four treatments, each with at least 10 replicates. (1) Intact leaf volatile compounds: odors from intact leaves of natural mulberry plants were collected *in situ* using oven bags. (2) Mechanically-damaged leaf volatile compounds: 30% of leaves on a mulberry plant were cut in half and volatiles were collected by encasing plants in oven bags. (3) Herbivore-induced leaf volatile compounds: ten *H. cunea* larvae were put into oven bags with mulberry plants. (4) Larvae: ten third-instar larvae were placed without leaves in oven bags.

For all four treatments, air was collected via an air sampler (QC-1, Beijing Municipal Institute of Labour Protection, Beijing, PRC) and 0.3 g of Super Q absorbent connected to the oven bags. Air flow was at 50 ml/min and air into the bags was purified by an activated carbon sphere on the inlet of the bags. Volatile samples were collected for 24 hours before elution with 2 ml of solvent (70% pentane and 30% diethyl ether), and a total of approximately 72l volume of air was captured through absorbent. Eluted samples were kept in a refrigerator at −20°C prior to subsequent analyses.

### Chemical analyses

Samples were analyzed by gas chromatography-mass spectrometry (GC-MS) using an Agilent 5973N (Santa Clara, CA, USA) mass selective detector coupled with an Agilent 6890N network GC system equipped with a quartz capillary column (DB-WAX, 60 m×0.25 mm×0.25 µm; J&W Scientific, Palo Alto, CA, USA). The column oven was maintained at 37°C for 2 min, increased to 150°C at 5°C/min, held for 2 min, increased to 200°C at 10°C/min, held for 5 min, and finally heated to 230°C at 20°C/min, and held for 1 min. The carrier gas was helium at a constant flow rate of 1 ml/min. The injection port was at 250°C with no split-flow.

### Electrophysiological analyses

Combined gas chromatography-flame ionization detection coupled with electroantennographic detection (GC-EAD) was used to identify compounds that were electrophysiologically active in male moths. The GC column and temperature program was identical to that of the GC-MS analysis above. Each antenna was prepared by cutting both extremes and immediately mounted between two Ag/AgCl electrodes filled with Kaissling saline (glucose 354 mmol/L, KCl 6.4 mmol/L, KH_2_PO_4_ 20 mmol/L, MgCl_2_ 12 mmol/L, CaCl_2_ 1 mmol/L, NaCl 12 mmol/L, KOH 9.6 mmol/L, pH 6.5). The electrode at the distal end of the antenna was connected via an interface box to a signal acquisition interface board (IDAC; Syntech, Kirchzarten, Germany) connected to a computer. EAD signals and flame ionization detector (FID) responses from the GC were simultaneously recorded by a computer using AutoSpike software (Syntech).

### Wind tunnel experiments

Wind tunnel experiments were conducted with 2–3 day old virgin male moths in a tunnel with a flying section of 90×90×240 cm (Heling Electronics Co. Ltd, Taiwan, China). Filtered air was blown by a centrifugal fan (Chongqing Dema Frequency Motor R & D Co. Ltd, Chongqing, China) at 30 cm/s. The outflowing air was aspired by another fan and released through a pipe. The flight section was lit diffusely from the top at 10 lux, and the room was kept at 23±2°C and 40–60% relative humidity. Experiments were carried out at the 6–7th hour of the dark phase [2].

Intact leaf volatile compounds, mechanically-damaged leaf volatile compounds, herbivory-induced volatile compounds, β-ocimene and cis-2-penten-1-ol were used in wind tunnel experiments. They were used individually as olfactory stimuli and also used as synergists coupled with 1/4 Nitolure synthetic pheromone lures for *H. cunea* (Nitto Denko, Osaka, Japan).

Gray rubber septa (The West Company, Phoenixville, PA) loaded with 100 µl solutions of compound samples were used in the experiments. β-ocimene and cis-2-penten-1-ol were at a dose of 1000 µg per septum [14]. Solvent and 1/4 pheromone lures for *H. cunea* were respectively used as a control in each group.

Olfactory stimuli were released from the center of the upwind end of the flight tunnel. Each compound was tested with 50 male moths, which were divided into 10 groups each with 5 individuals. Moths were released at the center of the downwind end in a small metal screen cage and behaviors were recorded for 5 min per moth. An orientation flight (more than 50 cm of tight zigzag flight to within 10 cm of the target) was recorded as positive [15]. Numbers of moths attracted were converted into a percentage and the effects of different compounds on moth attraction were tested using one-way ANOVA. Duncan's multiple-comparison test was used to compare mean differences between groups at *P* = 0.05. All analyses were performed in SPSS 16.0.

### Long-range field experiments

Gray rubber septa loaded with 100 µl solutions of the compounds (with a concentration of 10 mg/ml) were used in the field trials. After evaporation of the solvent, the rubber septa were wrapped in aluminum foil and stored at −20°C until used in field tests.

Field trapping experiments were executed from 3–14 September, 2011 in Laixi, Shandong Province, China, using the 11 active compounds and the headspace mixtures from mechanically- and herbivore-damaged leaves. Each compound was used with Nitolure synthetic pheromone lures for *H. cunea* in a single unitrap (Pherobio Technology Co., Beijing, China) at a dose of 1000 µg/septum. Nitolures alone were used as control.

Unitraps were hung 1.5–1.8 m above the ground and 50 m or more apart in poplar trees. Daily numbers of moths captured were transformed to log_10_ (n+1) prior to statistical analysis [16]. The effects of different compounds on the mean number of moths captured were tested using one-way ANOVA. LSD multiple-comparison test was used to compare mean differences between groups at *P* = 0.1. All analyses were performed in SPSS 16.0.

### Ethics Statement

All necessary permits were obtained for the described field studies from the Forest Pest Control and Quarantine Bureau of Shandong Province. These field studies did not involve endangered or protected species.

## Results

### Chemical and electrophysiological analyses

**Figure 1:**
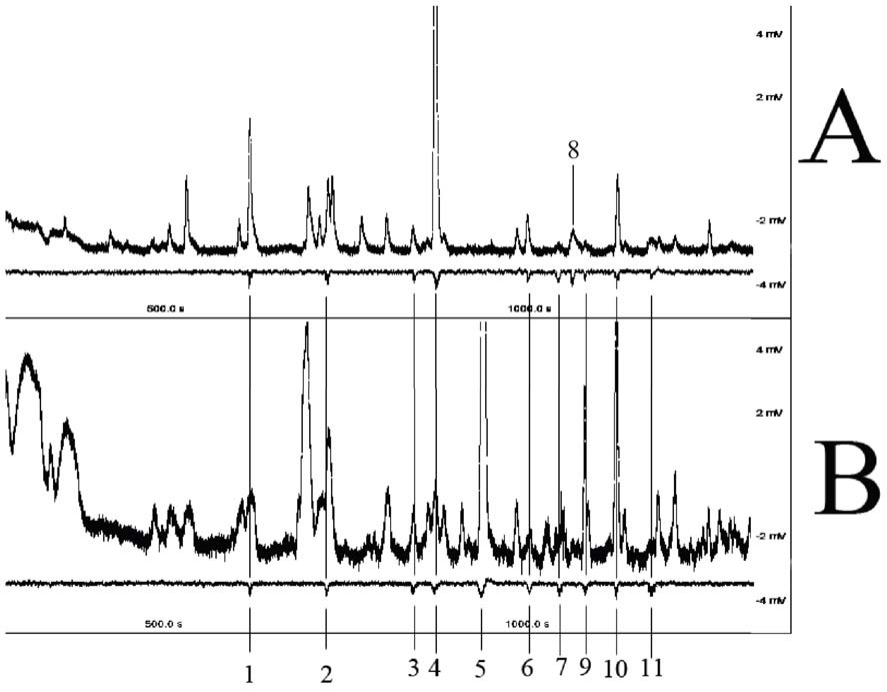
GC-EAD responses of *Hyphantria cunea* antennae to mulberry leaf volatiles. (A) Responses of one male moth to mechanically-damaged leaf volatiles. (B) Responses of one male moth to herbivore-damaged leaf volatiles. β-ocimene (5) was only detected from herbivore-damaged leaves (B), while cis-2-penten-1-ol (8) could be detected only in leaves that had not been eaten (A) and mechanical damage showed no influence on release of this compound. 1: hexanal; 2: cis-3-hexenal; 3: limonene; 4: trans-2-hexenal; 5: β-ocimene; 6: cyclohexanene; 7: 2,4-dimethyl-3-pentanol; 8: cis-2-penten-1-ol; 9: 6-methyl-5-hepten-2-one; 10: 4-hydroxy-4-methyl-2-pentanone; 11: trans-3-hexen-1-ol. CG-EAD: combined gas chromatography-flame ionization detection coupled with electroantennographic detection.

**Figure 2:**
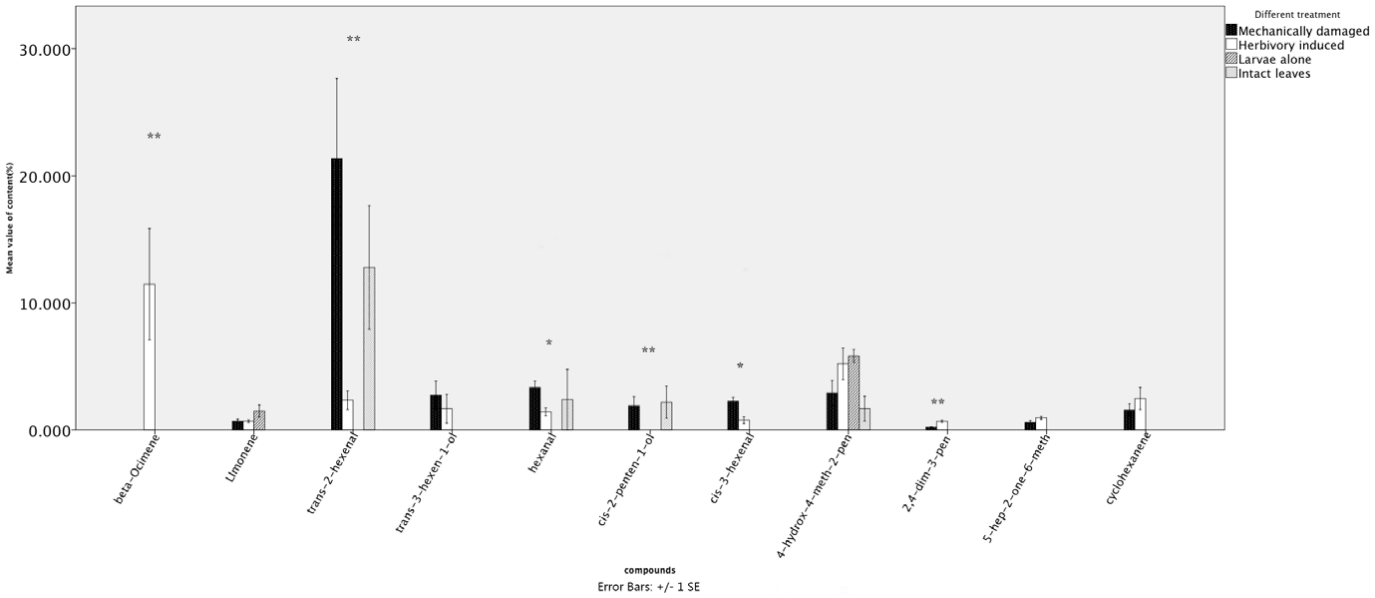
Mean values of bioactive components from intact, mechanically-damaged, herbivore-damaged mulberry leaves and *Hyphantria cunea* larvae. All 11 bioactive components were determined by gas chromatography-mass spectrometry (GC-MS) and combined gas chromatography-flame ionization detection coupled with electroantennographic detection (CG-EAD). Asterisks indicate significant differences between mechanically- and herbivore-damaged leaves for each compound (independent samples *t*-test; *, *P*<0.05 and **, *P*<0.01).

The GC-MS and GC-EAD analyses showed that 11 compounds elicited electrophysiological responses: hexanal, cis-3-hexenal, limonene, trans-2-hexenal, cyclohexanone, cis-2-penten-1-ol, 6-methyl-5-hepten-2-one, 4-hydroxy-4-methyl-2-pentanone, trans-3-hexen-1-ol, β-ocimene, and 2,4-dimethyl-3-pentanol (Figure 1). No unique volatiles were released by the larvae group (larvae alone). Although limonene and 4-hydroxy-4-methyl-2-pentanone could be detected from larvae, these compounds were also detected from mechanically-damaged leaves. Therefore, we concluded that these 11 active compounds were released by mulberry leaves. Only one compound, cis-2-penten-1-ol, was unique to leaves that had not suffered herbivore damage; it was detected in both intact and mechanically-damaged leaves. Interestingly, β-ocimene could be only detected from herbivore-damaged leaves and at high levels (with an average of 14% of total mass). Intact leaves released four active volatiles: trans-2-hexenal, hexanal, cis-2-penten-1-ol, and 4-hydroxy-4-methyl-2-pentanone. None of these four compounds differed between intact and mechanically-damaged leaves. Damage-induced volatiles that were not released by intact leaves, but could be detected from both mechanically- and herbivore-damaged leaves, were trans-3-hexen-1-ol, cis-3-hexenal, 6-methyl-5-hepten-2-one, 2,4-dimethyl-3-pentanol and cyclohexanene. Herbivory-influenced variation in each compound was tested with independent-samples *t*-tests (*P* = 0.05) between groups of mechanically- and herbivore-damaged leaves. Six compounds showed significant herbivory-influenced differences in production: β-ocimene, trans-2-hexenal, hexanal, cis-2-penten-1-ol, cis-3-hexenal and 2,4-dimethyl-3-pentanol (Figure 2). Concentrations of total bioactive compounds were at an average of 10.96 µg/µl and the average of total VOC mass captured by each absorbent was 21.92 mg.

### Wind tunnel experiments

**Figure 3:**
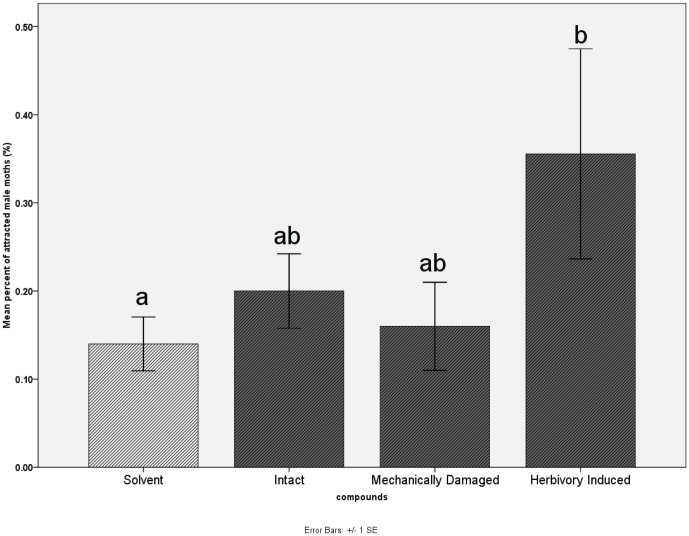
Attraction of male moths to volatile compounds from mulberry leaves in four treatments. Results from wind tunnel experiments using headspace absorbed volatile compounds. ‘Solvent’ was control, ‘intact’, ‘mechanically-damaged’ and ‘herbivory-damaged’ mulberry leaves were treatment groups. Intact and mechanically-damaged leaf volatiles did not attract male moths compared with solvent control. Herbivory-induced volatiles showed significant attractiveness to male moths (Duncan's multiple comparison tests, P<0.05).

**Figure 4:**
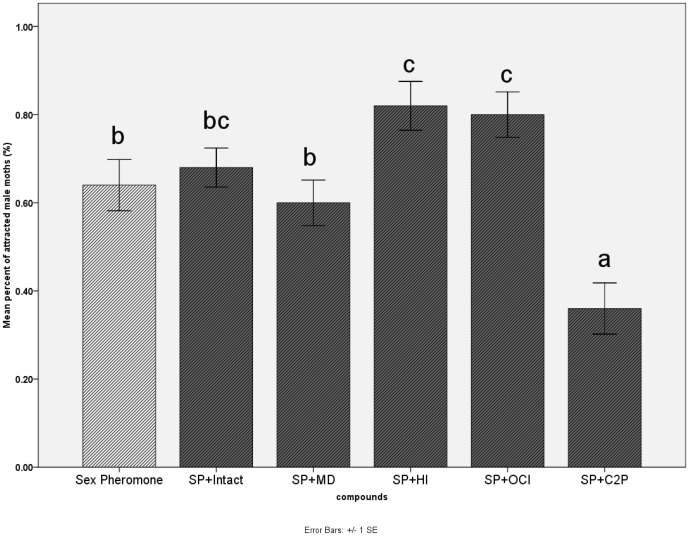
Attraction of male moths to volatile compounds combined with *H. cunea* sex pheromone lure. Results from wind tunnel experiments using headspace absorbed volatile compounds grouped into treatments: sex pheromone lure used as control; sex pheromone (SP) plus intact leaf volatiles; sex pheromone (SP) plus mechanically-damaged leaf volatiles (MD); sex pheromone (SP) plus herbivore-induced leaf volatiles (HI); sex pheromone (SP) plus β-ocimene; sex pheromone (SP) plus cis-2-penten-1-ol. Intact and mechanically-damaged leaf volatiles did not affect attraction of male moths to the sex pheromone. Herbivory-induced volatiles and β-ocimene significantly increased attraction of male moths to the sex pheromone, while cis-2-penten-1-ol significantly decreased attraction (Duncan's multiple comparison tests, P<0.05).

Intact leaf volatiles, mechanically-damaged leaf volatiles and herbivory-induced leaf volatiles were used alone as olfactory stimuli in wind tunnel experiments. Duncan's multiple-comparison test showed that herbivory-induced volatiles were significantly more attractive to male moths than the solvent control (Figure 3). Further wind tunnel experiments were conducted after combining a sex pheromone with each of the following compounds: intact leaf volatiles, mechanically-damaged leaf volatiles, herbivory-induced leaf volatiles, β-ocimene and cis-2-penten-1-ol. Male moth's attraction to the sex pheromone was significantly increased by herbivory-induced volatiles and β-ocimene, but significantly decreased by cis-2-penten-1-ol (Figure 4).

### Long-range field trapping

**Figure 5:**
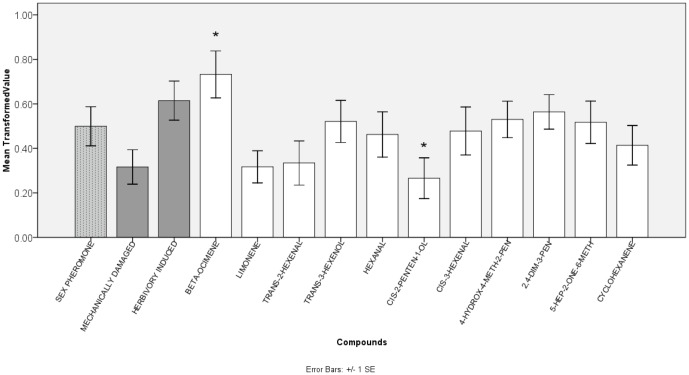
Long-range attractiveness of 13 volatiles and a sex pheromone to *Hyphantria cunea* in the field. Most compounds did not differ significantly from the sex pheromone control. β-Ocimene substantially increased the attractiveness of the sex pheromone, while cis-2-penten-1-ol substantially decreased its attractiveness (Duncan's multiple-comparison tests, *P*<0.1).

A total of 446 moths, all males, were captured in 28 unitraps. Numbers of moths captured by various compounds were compared. Duncan's multiple-comparison test showed that cis-2-penten-1-ol significantly lowered the numbers of moths captured, while β-ocimene significantly increased the numbers of moths captured relative to the sex pheromone control alone (Figure 5).

## Discussion

Green leaf volatiles are considered to be varieties of kairomones [17]. Many studies have indicated that olfactory reactions of male insects can be heavily affected by leaf volatiles [18]–[21]. When combined, botanical secondary substances and sex pheromones can attract *Helicoverpa armigera* moths over large distances [22]. Plant odors can also attract *Paralobesia viteana* and *Lobesia Botrana* moths in wind tunnel experiments [23], [24]. In the present study, herbivory-induced leaf volatiles were attractive to male *H. cunea* moths in wind tunnel experiments. Moreover, herbivory-induced volatiles and compounds like β-ocimene could significantly affect the attraction of moths to sex pheromones. However, when plant odors were used in the field, the majority, with the exception of β-ocimene and cis-2-penten-1-ol, did not significantly affect the attraction of moths to sex pheromones. These results suggest that effects of plant odors may be altered by volatiles emitted from plants in the field. Functions of plant volatiles could be more widespread and complicated in the natural environment. Although blends of mulberry leaf volatiles had significant effects on the attractiveness of the sex pheromone in the laboratory, some individual components did affect sex pheromone attractiveness even in the field. Compounds of VOCs of mulberry leaves, such as β-ocimene and cis-2-penten-1-ol, could potentially affect the location abilities of male *H. cunea* moths over a larger range than sex pheromones alone. That blends of VOCs showed no effect may be because the different functions of the individual components balanced one another out. Thus, botanical chemicals may have complex effects on this moth species.

The floral scent compound z-ocimene has also been reported to induce herbivory in a wide range of plants [25]–[28]. Non-specific storage of ocimene, because of its limited volatility, is a common phenomenon [29], and we found significant quantities of ocimene in the VOCs. Ocimene is known to activate defensive responses in *Arabidopsis thaliana* [27], [30] and functions as a volatile brood pheromone involved in social regulation in honey bee colonies [31]. Thus, ocimene may participate in a wide range of regulatory functions in ecosystems. Because ocimene is a taxonomically-widespread volatile that is induced by herbivory, attraction to it may offer *H. cunea* a broader range of host plants compared with other pests, which are repelled by herbivory-induced odors. However, herbivory-induced volatiles can also attract parasitic wasps that are known to attack *H. cunea*. This suggests that *H. cunea* is more likely to attack plants that have been fed on by other insects and are emitting ocimene, but that they endure a higher risk of being parasitized by natural enemies that are also attracted to those herbivory-damaged plants. We observed that parasitism rates of *H. cunea* pupae were remarkably high in the wild (personal observation), which supports the assumption above. Conversely, cis-2-penten-1-ol decreased the trapping rate of the sex pheromone lure. This compound is commonly found in plant oils [32] and has been suggested to contribute to the development of unpleasant fishy and rancid odors [33]. Peppermint oil, which also contains cis-2-penten-1-ol, can deter insects [34]. The presence of this component only in volatiles emitted from leaves that were not herbivore damaged could explain the reduction in moth captures by the volatiles from mechanically-damaged leaves combined with the sex pheromone.

Because only male moths were captured in our experiments, we suggest that the sex pheromone repelled female moths, as has been shown in other Lepidoptera species [35]. We speculate that mulberry VOCs were apparently not sufficiently attractive to *H. cunea* females to overcome the possible repellency of the sex pheromone.

We conclude that the herbivory-induced leaf volatiles of *M. alba* can attract *H. cunea* male moths in wind tunnel experiments. Furthermore, a blend of head space absorbed herbivory-induced volatiles and some components of volatiles, including β-ocimene and cis-2-penten-1-ol, could affect attraction of *H. cunea* sex pheromones. A blend of these volatile compounds could have more complex functions in nature. Mulberry trees contain compounds such as cis-2-penten-1-ol, which act as a natural shield against insects. However, after attack, herbivore-induced volatiles such as β-ocimene may attract male *H. cunea* by increasing its sensitivity towards sex pheromones. The invasion of China by this insect may differ from the movement patterns of local species because of their attraction to herbivore-induced compounds.
